# Supplementary material for: Demand forecasting for platelet usage: From univariate time series to multivariable models
Source: PLoS One. 2024 Apr 23;19(4):e0297391. doi: 10.1371/journal.pone.0297391 (PMC11037532; doi:10.1371/journal.pone.0297391)
Supplement: S1 Appendix — (PDF) [file pone.0297391.s001.pdf]

Supporting Information

S1 Appendix

Table S1 gives the selected predictors using lasso regression. Considering the coefficients for the predictors and their corresponding confidence intervals in Table S1, and based on [1], variables that have a coefficient of zero and confidence intervals that are symmetric around zero are candidates to be eliminated. As we can see from Table S1, abnormal_plt has the highest coefficient. The predictors abnormal_hb and abnormal_redcellwidth can be considered as two other important lab tests for forecasting the demand. Day of the week, last week's platelet usage and yesterday's platelet usage also have notable impact on the platelet demand. As we can see in Table S1, unexpectedly, some of the predictors have a negative coefficient in the demand forecasting model. The reason is that, as we can see from Fig 8, there are high correlations among the predictors that result in interactions among the model predictors, which may cause multicollinearity issues. Specifically, the predictors abnormal_hb, abnormal_INR, abnormal_hematocrit, and abnormal_MPV are correlated with abnormal_plt. The predictors abnormal_hematocrit and abnormal_hb also have high correlations with most of the other abnormal laboratory test results.

Table S1. Predictors and their corresponding coefficients for lasso regression

**predictors**	**Coefficients**	95% **Confidence Interval**
abnormal_ALP	-0.02	(-0.08 , 0.04)
abnormal_MPV	0.01	(-0.06 , 0.11)
abnormal_hematocrit	0.00	(-0.11 , 0.14)
abnormal_PO2	-0.11	(-0.19 , 0.00)
abnormal_creatinine	0.03	(-0.03 , 0.11)
abnormal_INR	0.06	(-0.02 , 0.22)
abnormal_MCHb	-0.03	(-0.10 , 0.04)
abnormal_MCHb_conc	-0.03	(-0.10 , 0.04)
abnormal_hb	0.05	(-0.04 , 0.19)
abnormal_mcv	-0.03	(-0.11 , 0.04)
abnormal_plt	0.23	(0.02 , 0.36)
abnormal_redcellwidth	0.07	(0.00 , 0.15)
abnormal_wbc	-0.02	(-0.09 , 0.03)
abnormal_ALC	0.01	(-0.05 , 0.08)
location_GeneralMedicine	-0.11	(-0.21 , 0.00)
location_Hematology	0.04	(-0.02 , 0.16)
location_IntensiveCare	0.05	(-0.01 , 0.15)
location_CardiovascularSurgery	0.04	(-0.03 , 0.11)
location_Pediatric	0.04	(-0.02 , 0.10)
Monday	0.07	(0.00 , 0.16)
Tuesday	0.07	(0.00 , 0.14)
Wednesday	0.00	(-0.04 , 0.07)
Thursday	0.01	(-0.03 , 0.09)
Friday	-0.39	(-0.46 , -0.31)
Saturday	-0.31	(-0.39 , -0.23)
Sunday	0.10	(0.03 , 0.18)
lastWeek_Usage	0.12	(0.05 , 0.19)
yesterday_Usage	0.10	(0.02 , 0.17)
yesterday_ReceivedUnits	0.06	(0.00 , 0.14)

The coefficients for lab tests are high. This is consistent with the observation that the lab test results are significant indicators for platelet transfusion. The predictors

abnormal_plt, abnormal_hb, abnormal_ALC and abnormal_wbc have higher coefficients and consequently higher impact on platelet demand. For day of the week, Friday and Saturday have negative coefficients due to the fact that they cover the weekend (Friday: -0.39 and Saturday: -0.31). For hospital census data, except for location_GeneralMedicine, all the coefficients are in a similar range to the lab tests.

References

1. Ranstam J. Why the P-value culture is bad and confidence intervals a better alternative. Osteoarthritis and Cartilage. 2012;20(8):805–808.
